# Supplementary material for: Entropy is an important design principle in the photosystem II supercomplex
Source: Proc Natl Acad Sci U S A. 2025 Mar 19;122(12):e2426331122. doi: 10.1073/pnas.2426331122 (PMC11962461; doi:10.1073/pnas.2426331122)
Supplement: Supplementary file 1 — Appendix 01 (PDF) [file pnas.2426331122.sapp.pdf]

# Supporting Information for

Entropy is an Important Design Principle in the Photosystem II Supercomplex

Johanna L. Hall[a,b,c], Shiun-Jr Yang[a,b,c,f], David T. Limmer [a,c,d,e], and Graham R. Fleming[a,b,c,*]

[a]Department of Chemistry, University of California, Berkeley, Berkeley, CA 94720.
[b]Molecular Biophysics and Integrated Bioimaging Division, Lawrence Berkeley National Laboratory, Berkeley, CA 94720.
[c]Kavli Energy Nanoscience Institute at Berkeley, Berkeley, CA 94720.
[d]Chemical Sciences Division, Lawrence Berkeley National Laboratory, Berkeley, CA 94720.
[e]Materials Sciences Division, Lawrence Berkeley National Laboratory, Berkeley, CA 94720.
[f]Current Affiliation: Department of Chemistry, Massachusetts Institute of Technology, Cambridge, MA 02139.

[*]**Corresponding Author:** Graham R. Fleming
Hildebrand Hall 221, University of California, Berkeley, Berkeley, CA 94720
(510) 520-4220
grfleming@lbl.gov

**This PDF file includes:**

Supporting text
Figures S1 to S11
Table S1
SI References

## Supporting Information Text

### Construction of the Kinetic Rate Matrix.

The kinetic rate matrices used in this study have been published elsewhere[1,2]. Briefly, they were constructed following the methods outlined by Bennett et al.[3], and are based on the structures obtained by Wei et al.[4] and Su et al.[5] Semi-empirical Hamiltonians for the reaction center[6], CP43[7,8], CP47[7], CP29[9], and LHCII[10] subunits were obtained from literature. These Hamiltonians include both the site energies and inter-pigment coupling values for each chlorophyll in the subunit. Because such Hamiltonians are not available for the CP26 and CP24 subunits, the CP29 and LHCII Hamiltonians, with extra chlorophyll molecules removed, were respectively used due to their structural and spectral homology.

The coupling values between pigments in different protein subunits were calculated using the TrEsp method[11]. The equations and parameters used for the spectral density, transition dipole moments, and inhomogeneous linewidths are the same as used by Leonardo et al.[1] and Yang et al.[2] Due to fluctuations in site energy, a random number is added to the site energy of each chlorophyll, based on the inhomogeneous broadening linewidth of that site. All chlorophylls are separated into domains, which are defined based on the coupling strength and degree of exciton delocalization across pigments. To be in the same domain, two pigments must have a calculated coupling strength of at least $V_{\mathrm{cutoff}} = 15\mathrm{cm}^{-1}$[1–3]. For each inhomogeneous realization of the random modulation of the site energies, coupling strengths below the cutoff are removed and the transformation matrix is calculated by diagonalizing the site-basis Hamiltonian. This matrix reveals the excitonic overlap between sites. If the overlap $S$ between sites $\mu$ and $\gamma$ satisfies $S_{\mu,\gamma} > 0.1$ for at least 50% of all 500 inhomogeneous realizations, then the two sites are defined as being in the same domain[1–3].

To compute energy transfer rates, the Hamiltonian is first block-diagonalized within each defined domain. For states in the same domain, we assume strong electronic coupling where energy transfer is driven by weak electron-phonon interactions. To compute intra-domain energy transfer rates, we apply modified Redfield theory[1–3,12]. For states in different domains, we assume strong electron-phonon coupling where energy transfer is driven by electrostatic interactions. To compute inter-domain energy transfer rates, we apply generalized Forster theory[1–3,12]. We again apply a random number to perturb the site energies and calculate the rate matrix for these different inhomogeneous realizations 500 times. Each rate matrix satisfies detailed balance with respect to the site energies. To obtain the final rate matrix, we average each realization.

### Simulation Details.

The trap state in the reaction center was set to an energy of 13,500 $cm^{-1}$, approximately 1,000 $cm^{-1}$ less than the lowest-energy state in PSII (14,491 $cm^{-1}$). One trap state was defined for each PSII monomer, located equidistant between $Chl_{D1}$ and $Pheo_{D1}$. All calculations were performed at 300K.

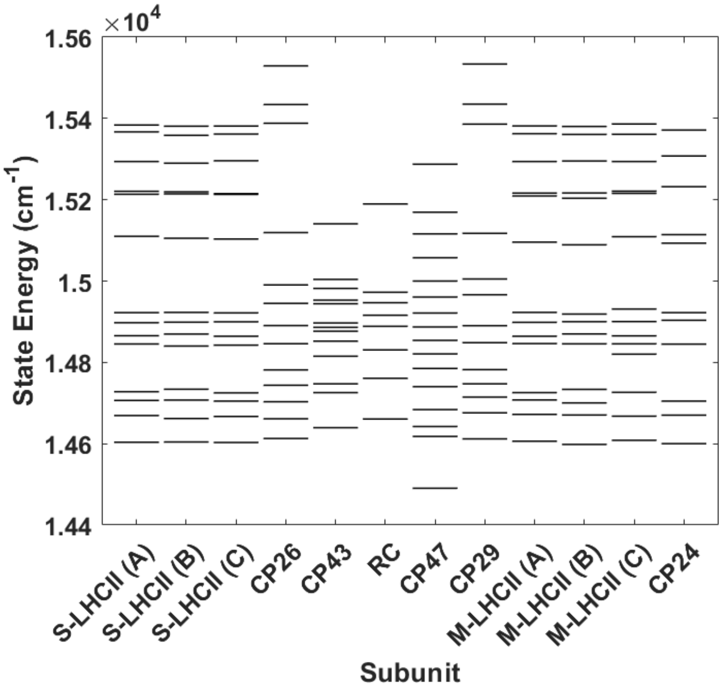


**Fig. S1**. Energy levels for each exciton in the $C_2S_2M_2$-type PSII supercomplex based on the semi-empirical Hamiltonians reported in literature[6,7,9,10,13].

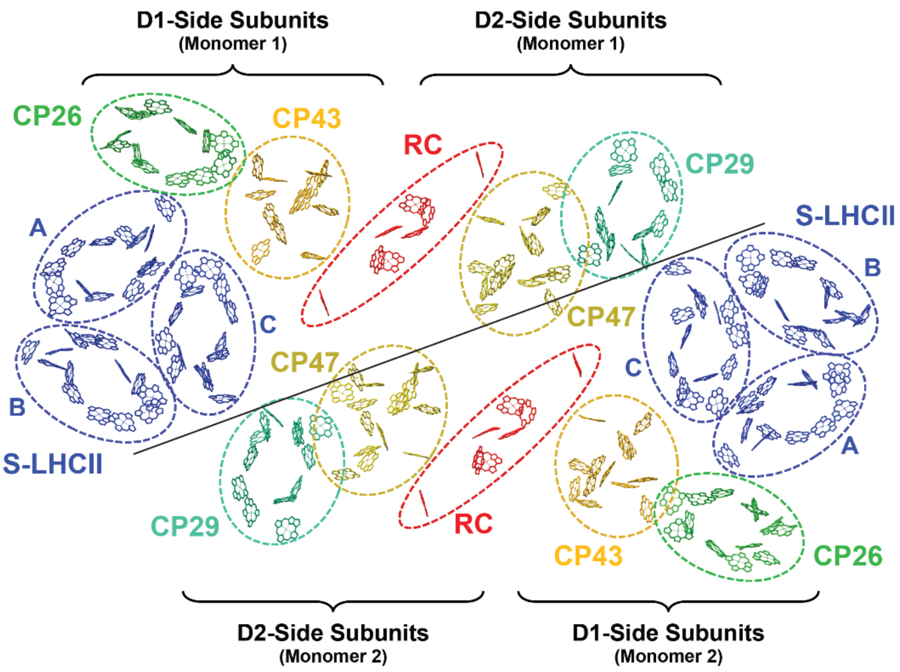


**Fig. S2.** The pigment arrangement of the $C_2S_2$-type PSII supercomplex with protein subunits labeled (PDB: 3JCU)[4]. The solid line marks the separation of the two monomers.

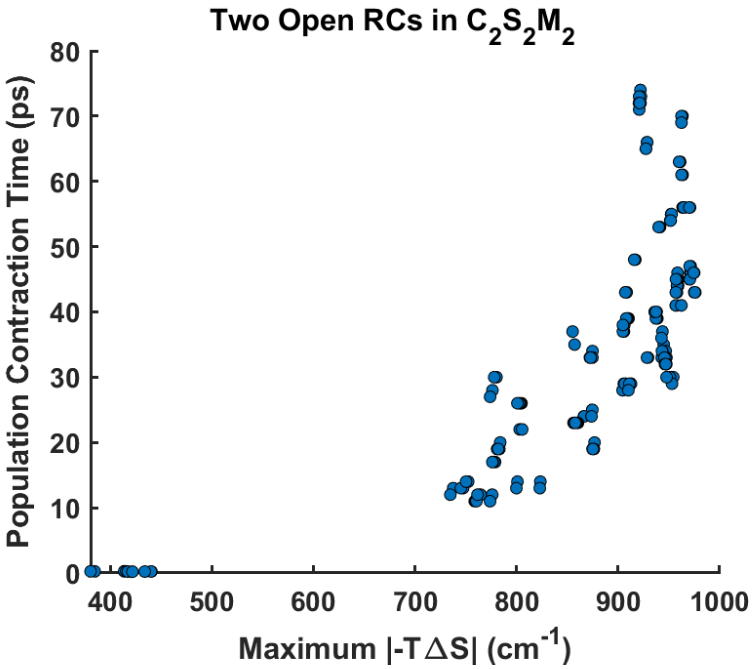


**Fig. S3**. Correlation between the maximum entropy and the population contraction time, or the time at which this entropy maximum occurs, for all initial excitation locations in the $C_2S_2M_2$-type PSII supercomplex.

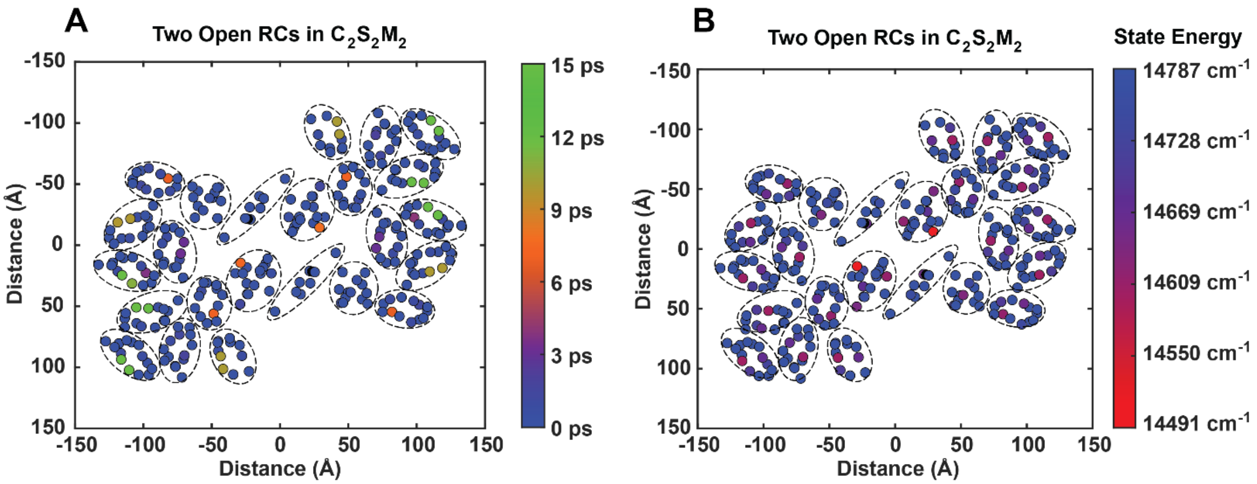


**Fig. S4**. (A) The time when the enthalpy term is at a maximum for each initially-excited state and (B) the energy of the initially-excited states in the $C_2S_2M_2$-type PSII supercomplex, cut off at a maximum of 14,800 cm$^{-1}$ and projected onto the site basis.

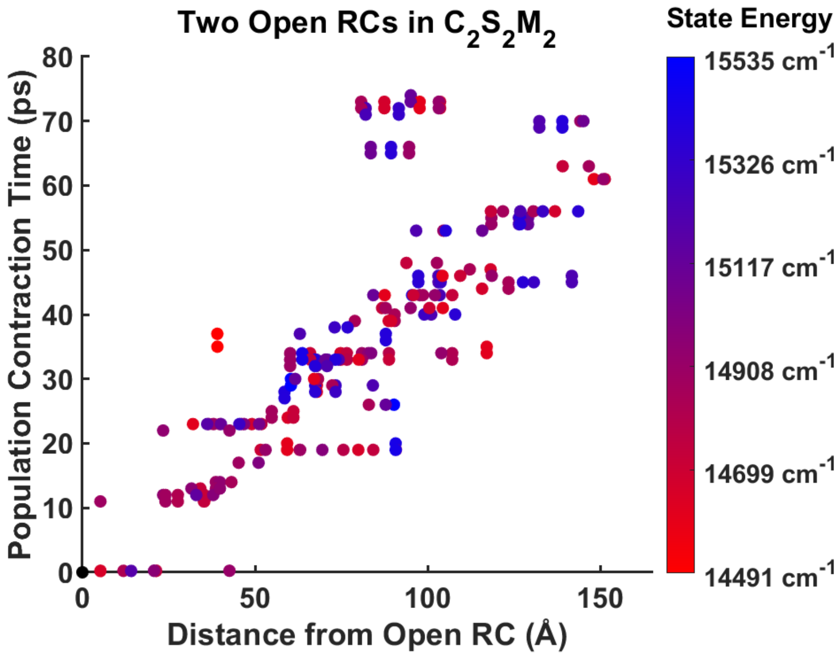


**Fig. S5**. The correlation between a state's distance from the closest open RC and its population contraction time in the $C_2S_2M_2$-type PSII supercomplex, color coded by the state energy.

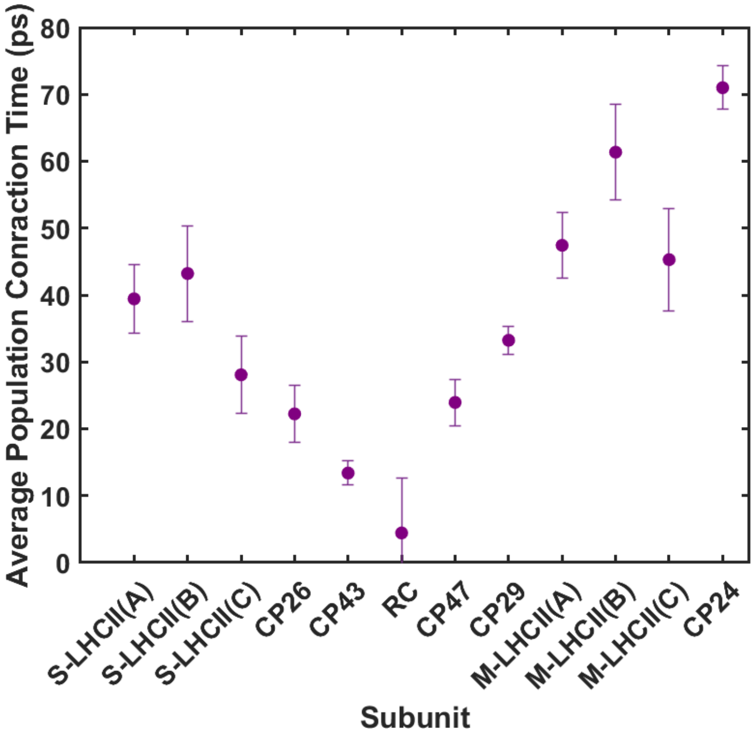


**Fig. S6**. Subunit-averaged population contraction times for each initial excitation in the $C_2S_2M_2$-type PSII supercomplex. The plot omits results for monomer 2 subunits due to their high similarity to monomer 1 subunits.

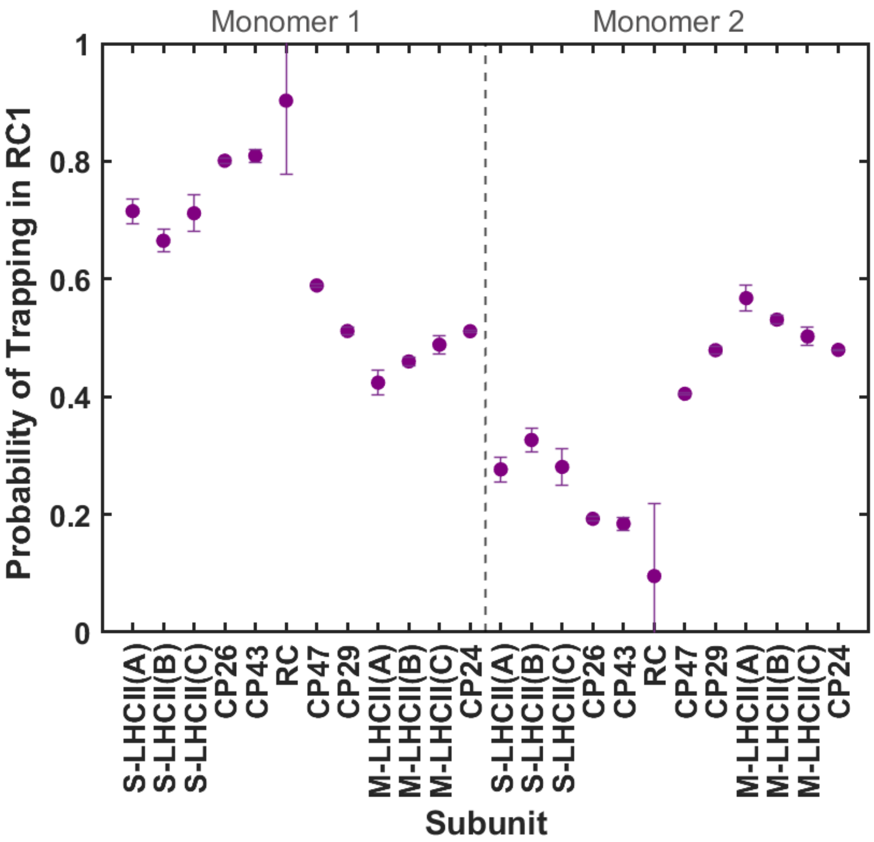


**Fig. S7.** Subunit-averaged probability of excitations being trapped in the monomer 1 reaction center when both reaction centers are open, for each initial excitation in the $C_2S_2M_2$-type PSII supercomplex.

.

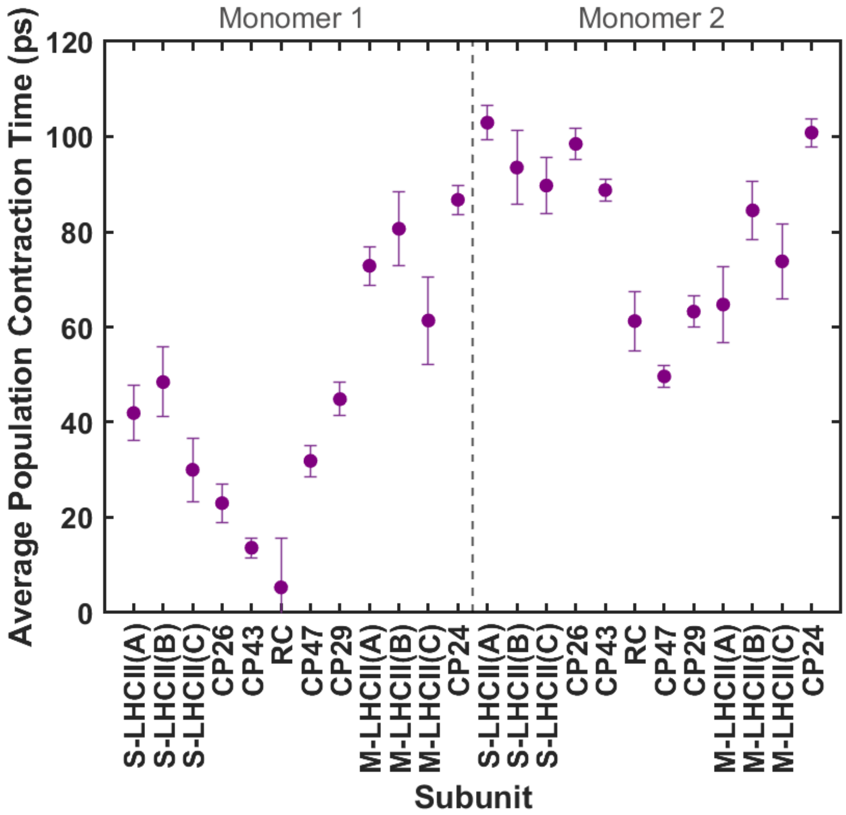


**Fig. S8**. Subunit-averaged population contraction time when the monomer 2 reaction center is closed, for each initial excitation in the $C_2S_2M_2$-type PSII supercomplex.

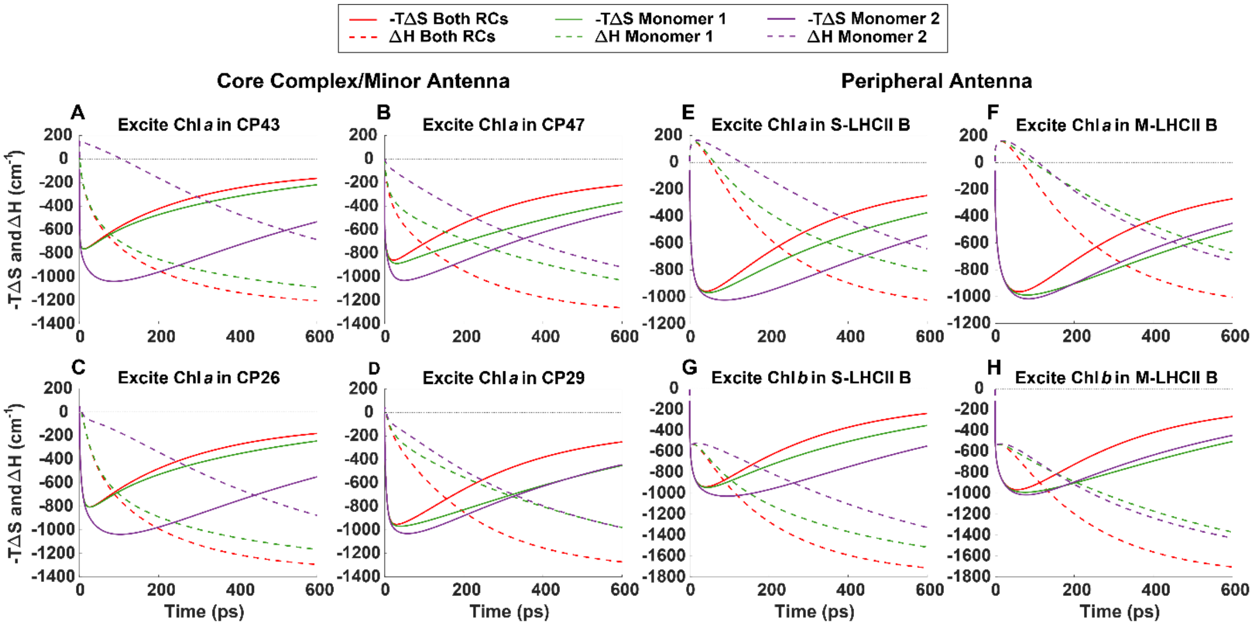


**Fig. S9.** Entropy (solid line) and enthalpy (dashed line) components of the free energy change in time for initial excitations at selected states in the core (A-B), minor antenna (C-D), and peripheral antenna (E-H) complexes. Red lines indicate excitation in the $C_2S_2M_2$ complex with two open RCs, green lines indicate excitation in the monomer 1 side of the complex when only the monomer 1 RC is open, and purple lines indicate excitation in the monomer 2 side of the complex when only the monomer 1 RC is open. In order of A-E, excitations are localized in Chl *a* 509 in CP43, Chl *a* 610 in CP47, Chl *a* 604 in CP26, Chl *a* 604 in CP29, Chl *a* 610 in S-LHCII (B), Chl *a* 610 in M-LHCII (B), Chl *b* 609 in S-LHCII (B), and Chl *b* 609 in M-LHCII (B).

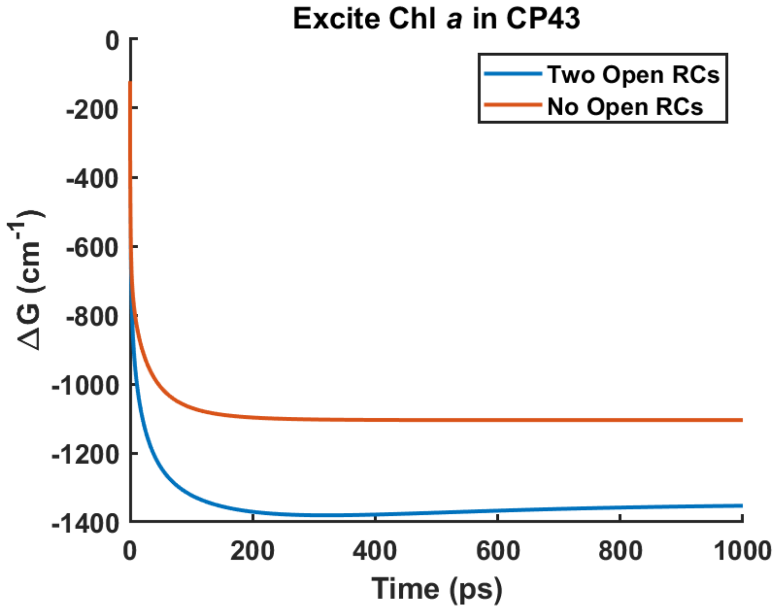


**Fig. S10**. The total free energy change in time for an initial excitation localized in Chl *a* 509 in CP43, for the $C_2S_2M_2$-type PSII supercomplex with two open RCs (blue) and no open RCs.

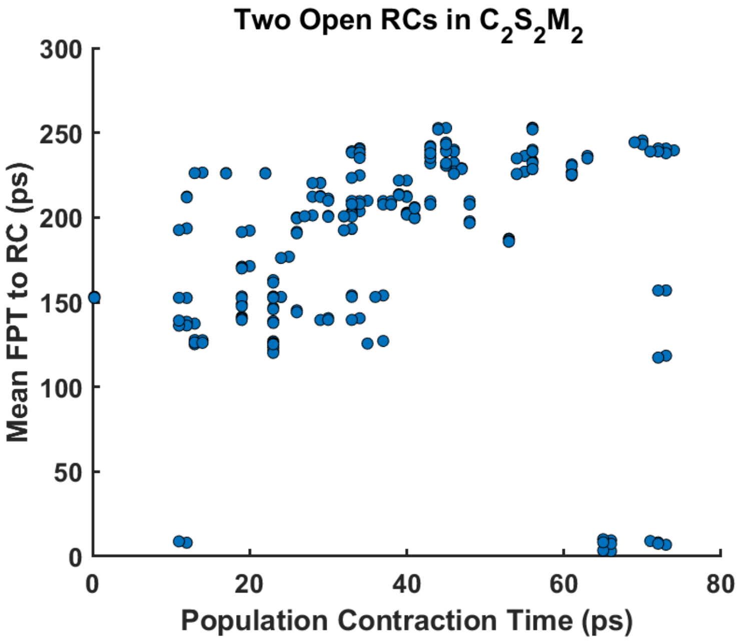


**Fig. S11**. Correlation between the mean first passage time (FPT) to the reaction center and the population contraction time for all initial excitation locations in the $C_2S_2M_2$-type PSII supercomplex.

**Table S1.** Percent change in the population contraction time upon closure of the monomer 2 RC for selected excitations in the $C_2S_2$ and $C_2S_2M_2$ PSII supercomplexes.

| Excitation Location | Supercomplex Type | Contraction Time (ps): Both RCs Open | | Contraction Time (ps): Only Monomer 1 RC Open | | Monomer 1 % Change Two to One Open RC | Monomer 2 % Change Two to One Open RC |
|---|---|---|---|---|---|---|---|
| | | Excite Monomer 1 | Excite Monomer 2 | Excite Monomer 1 | Excite Monomer 2 | | |
| Chl *a* in CP43 | $C_2S_2$ | 10 | 10 | 11 | 58 | 10.0 | 480.0 |
| | $C_2S_2M_2$ | 12 | 11 | 12 | 87 | 0.0 | 690.9 |
| Chl *a* in CP47 | $C_2S_2$ | 12 | 12 | 18 | 42 | 50.0 | 250.0 |
| | $C_2S_2M_2$ | 23 | 23 | 31 | 49 | 34.8 | 113.0 |
| Chl *a* in CP26 | $C_2S_2$ | 24 | 23 | 24 | 76 | 0.0 | 230.4 |
| | $C_2S_2M_2$ | 26 | 26 | 26 | 103 | 0.0 | 296.2 |
| Chl *a* in CP29 | $C_2S_2$ | 30 | 30 | 38 | 56 | 26.7 | 86.7 |
| | $C_2S_2M_2$ | 30 | 30 | 40 | 58 | 33.3 | 93.3 |
| Chl *a* in S-LHCII | $C_2S_2$ | 42 | 42 | 45 | 101 | 7.1 | 140.5 |
| | $C_2S_2M_2$ | 41 | 41 | 48 | 87 | 17.1 | 112.2 |
| Chl *b* in S-LHCII | $C_2S_2$ | 39 | 39 | 41 | 99 | 5.1 | 153.8 |
| | $C_2S_2M_2$ | 40 | 40 | 44 | 94 | 10.0 | 135.0 |
| Chl *a* in M-LHCII | $C_2S_2M_2$ | 61 | 61 | 80 | 84 | 31.1 | 37.7 |
| Chl *b* in M-LHCII | $C_2S_2M_2$ | 56 | 56 | 76 | 79 | 35.7 | 41.1 |

**SI References**


1. Leonardo, C. *et al.* Bidirectional Energy Flow in the Photosystem II Supercomplex. *J. Phys. Chem. B* **128**, 7941–7953 (2024).
2. Yang, S.-J., Wales, D. J., Woods, E. J. & Fleming, G. R. Design principles for energy transfer in the photosystem II supercomplex from kinetic transition networks. *Nat Commun* **15**, 8763 (2024).
3. Bennett, D. I. G., Amarnath, K. & Fleming, G. R. A Structure-Based Model of Energy Transfer Reveals the Principles of Light Harvesting in Photosystem II Supercomplexes. *J. Am. Chem. Soc.* **135**, 9164–9173 (2013).
4. Wei, X. *et al.* Structure of spinach photosystem II–LHCII supercomplex at 3.2 Å resolution. *Nature* **534**, 69–74 (2016).
5. Su, X. *et al.* Structure and assembly mechanism of plant $C_2$ $S_2$ $M_2$ -type PSII-LHCII supercomplex. *Science* **357**, 815–820 (2017).
6. Raszewski, G., Saenger, W. & Renger, T. Theory of Optical Spectra of Photosystem II Reaction Centers: Location of the Triplet State and the Identity of the Primary Electron Donor. *Biophysical Journal* **88**, 986–998 (2005).
7. Raszewski, G. & Renger, T. Light Harvesting in Photosystem II Core Complexes Is Limited by the Transfer to the Trap: Can the Core Complex Turn into a Photoprotective Mode? *J. Am. Chem. Soc.* **130**, 4431–4446 (2008).
8. Müh, F., Madjet, M. E.-A. & Renger, T. Structure-based simulation of linear optical spectra of the CP43 core antenna of photosystem II. *Photosynth Res* **111**, 87–101 (2012).
9. Mascoli, V., Novoderezhkin, V., Liguori, N., Xu, P. & Croce, R. Design principles of solar light harvesting in plants: Functional architecture of the monomeric antenna CP29. *Biochimica et Biophysica Acta (BBA) - Bioenergetics* **1861**, 148156 (2020).
10. Novoderezhkin, V., Marin, A. & Van Grondelle, R. Intra- and inter-monomeric transfers in the light harvesting LHCII complex: the Redfield–Förster picture. *Phys. Chem. Chem. Phys.* **13**, 17093 (2011).
11. Madjet, M. E., Abdurahman, A. & Renger, T. Intermolecular Coulomb Couplings from Ab Initio Electrostatic Potentials: Application to Optical Transitions of Strongly Coupled Pigments in Photosynthetic Antennae and Reaction Centers. *J. Phys. Chem. B* **110**, 17268–17281 (2006).
12. Yang, M. & Fleming, G. R. Influence of phonons on exciton transfer dynamics: comparison of the Redfield, Förster, and modified Redfield equations. *Chemical Physics* **275**, 355–372 (2002).
13. Ghazy, A., Safdar, M., Lastusaari, M., Savin, H. & Karppinen, M. Advances in upconversion enhanced solar cell performance. *Solar Energy Materials and Solar Cells* **230**, 111234 (2021).
